# The MRGPRX2-substance P pathway regulates mast cell migration

**DOI:** 10.1016/j.isci.2024.110984

**Published:** 2024-09-16

**Authors:** Peter W. West, Jérémy Chéret, Rajia Bahri, Orsolya Kiss, Zining Wu, Colin H. Macphee, Silvia Bulfone-Paus

**Affiliations:** 1 Lydia Becker Institute of Immunology and Inflammation, School of Biological Sciences, Faculty of Biology, Medicine and Health, University of Manchester, Manchester Academic Health Science Centre, Manchester, UK; 2 Dr. Phillip Frost Department of Dermatology and Cutaneous Surgery, University of Miami Miller School of Medicine, Miami, FL, USA; 3 CUTANEON- Skin & Hair Innovation, Hamburg, Berlin, Germany; 4 GSK, 1250 South Collegeville Road, Collegeville, PA 19426, USA

**Keywords:** Immunology, Molecular biology, Cell biology

## Abstract

Mast cells (MCs) are tissue-resident immune cells known to degranulate in response to FcεRI crosslinking or MRGPRX2 engagement. MCs are found close to nerves, but the mechanisms that regulate this privileged localization remain unclear. Here, we investigated MRGPRX2 expression patterns and specific activities in MCs. We show that MRGPRX2 expression is heterogeneous in human MC (hMC) progenitors and mature MCs. Substance P (SP) is a rapid and specific activator of MRGPRX2, and long-term supplementation of MCs with SP expands MRGPRX2-expressing cells.

While high concentrations of SP induce rapid MC degranulation, low concentrations prompt immature MC chemotaxis. Lastly, we demonstrate that in inflammatory skin conditions like psoriasis, the number of MRGPRX2^+^ MCs is increased, and during *in vitro* skin reinnervation, MRGPRX2^+^ MCs preferentially reside in proximity to and migrate toward SP^+^ nerve fibers (NFs). This indicates that SP-MRGPRX2 signaling defines MC positioning and relocation within tissues and promotes immune cell-NF communication.

## Highlights

• MRGPRX2 expression is heterogeneous in human mast cells at all stages of maturation
• Low concentrations of substance P induce immature mast cell chemotaxis
• The number of MRGPRX2+ mast cells is increased in skin inflammation
• MRGPRX2^+^ mast cells reside in proximity to and migrate toward substance P^+^ nerve fibers

## Graphical abstract

**Figure:**
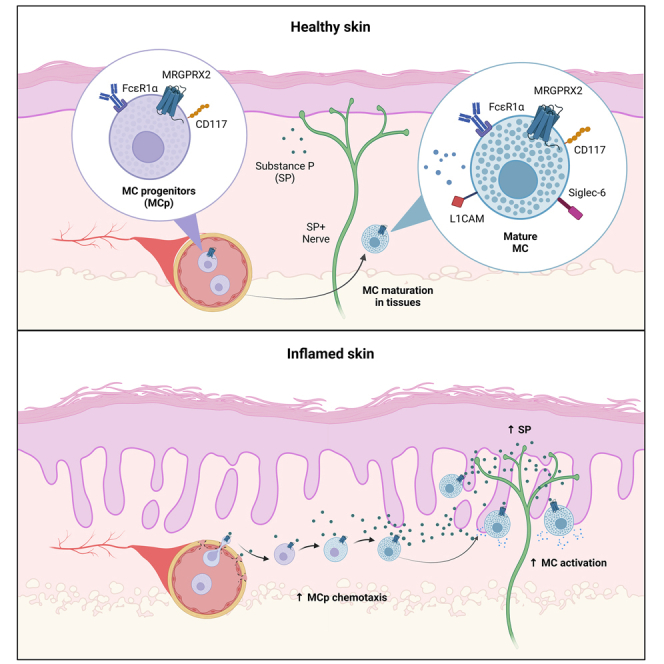

## Introduction

Mast cells (MCs) are long-lived, tissue-resident cells that, in humans and rodents, are recruited both during early development from the yolk sac and later in life from hematopoietic bone marrow progenitors in a tissue-specific pattern.^1^^,^^2^ Classically, MCs are activated by allergens and IgE-mediated crosslinking of the high-affinity IgE receptor (FcεRI) complex. However, it is well established that numerous IgE-independent pathways, such as the engagement of G protein-coupled receptors (GPCR), can trigger MC degranulation. Among them are the complement receptors^3^ and the recently discovered Mas-related G protein-coupled receptor b2 (Mrgprb2),^4^ and the human ortholog Mas-related G protein-coupled receptor X2^5^ (MRGPRX2) reviewed by Gour and Dong.^6^

MRGPRX2 is predominantly expressed by connective tissue MCs (fat and skin MCs) that express both tryptase and chymase in their granules and reside in skin and synovial fluid.^7^^,^^8^ MRGPRX2 binds several different ligands, called basic secretagogues, like proadrenomedullin N-terminal peptide (PAMP-12 and PAMP-20), antimicrobial peptides (cathelicidin LL-37 and β-defensins), cortistatin-14, venoms, and substance P (SP).^6^^,^^9^^,^^10^^,^^11^^,^^12^^,^^13^ The latter is involved in MC-mediated neurogenic inflammation, where low micromolar concentrations of synthetic SP can induce wheal and flare reactions in human skin.^14^

Since its identification, MRGPRX2 has been shown to contribute to pseudoallergic reactions,^4^ cutaneous allergic drug reactions, inflammatory pain,^15^ and non-histaminergic itch.^16^ It has also been implicated in inflammatory diseases such as atopic dermatitis, psoriasis,^17^ asthma,^18^ and drug allergies.^19^

In skin inflammation, steady-state, self-renewing, stable clonal colonies of MCs are disrupted by the influx of progenitors from the circulation, establishing a new MC density set-point.^20^ The movement of MCs and their progenitors to and within barrier tissues could play a crucial role in both short-term inflammatory reactions and long-term tissue homeostasis. Notably, mice lacking Mrgprb2 show reduced recruitment of MCs during inflammation.^15^ The extent to which the SP-MRGPRX2 axis controls MC activation and favors MC recruitment to sensory nerve fibers (NFs) or orchestrates MC relocation in the inflamed tissue remains to be explored.

## Results

### MRGPRX2 expression is highly heterogeneous between donors during hMC differentiation and heterogeneity is maintained in mature MCs

**Figure 1:**
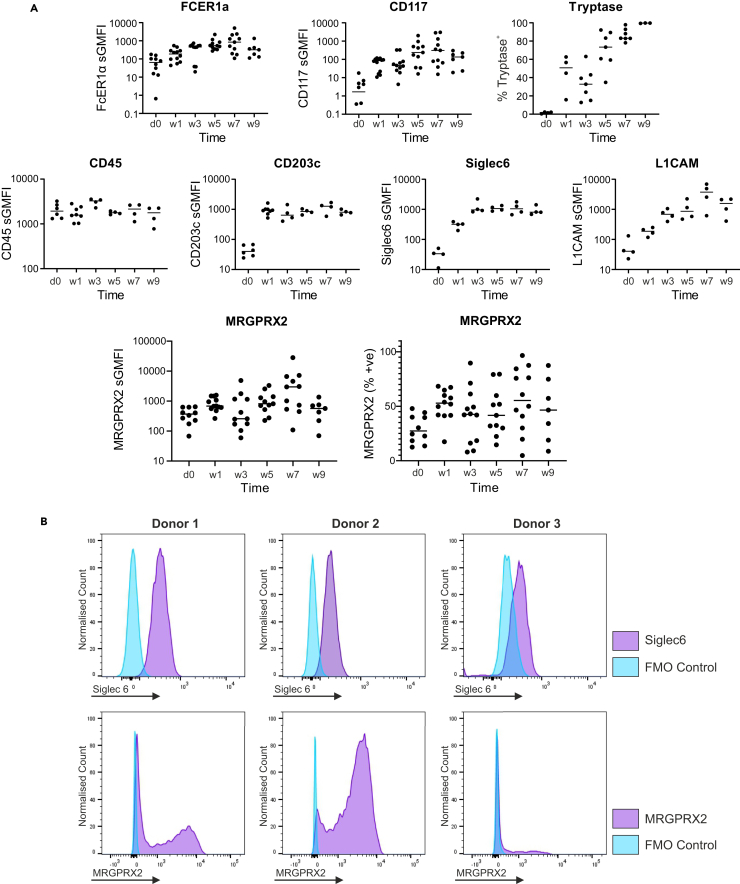
MRGPRX2 expression is highly heterogeneous between donors during human mast cell differentiation and heterogeneity is maintained in mature mast cells (A) Expression of MC markers and MRGPRX2 during *in vitro* differentiation of peripheral blood derived human MCs. Flow cytometric analysis using fluorescently labeled antibodies to quantify marker expression. Data are specific geometric mean fluorescence (sGMFI) (specific antibody – control values) or % of positive cells (percentage of cells with fluorescent values above control) for *n* = 3–12 separate donors (as shown) at day 0, 1, 3, 5, 7, and 9 weeks. (B) MRGPRX2 heterogeneity in comparison to Siglec6. Histograms showing Siglec6 or MRGPRX2 fluorescence intensity (x axis) vs. cell count (y axis) indicative of marker expression in three separate donors at maturity. Siglec6 fluorescence is shown in the top panels with homogenous populations of immuno-positive cells (purple) vs. control (turquoise). MRGPRX2 expression is shown in the lower panels with heterogeneous numbers of cells showing MRGPRX2-specific fluorescence intensity (purple) greater than control (turquoise).

MC progenitors express CD117/KIT and FcεRI, the best-known activating receptors in MCs. While IgE engages the FcεRI, MRGPRX2 activation is IgE-independent.^21^ It is unclear at which stage of MC differentiation MRGPRX2 is expressed at the membrane. Thus, MRGPRX2 expression in human MCs (hMCs) derived from blood progenitors was examined at different time points during differentiation. Cells freshly isolated by CD117 magnetic selection, negative for hematopoietic lineage markers (Lin-, data not shown), and positive for CD45 were investigated. These cells exhibited expression of FcεRIα and CD117 (Figure 1A), indicating a population of MC progenitors. Although clearly positive, CD117 appeared to be lower at d0 compared to other time points. This may be due to epitope masking by antibodies used for magnetic isolation. At this time (d0), CD45^+^ FcεRIα^+^ CD117^+^ cells did not express tryptase, and had very low levels of CD203c, CD327/Siglec6, and CD171/L1CAM. MRGPRX2 was expressed in variable amounts, with specific mean fluorescence above that of the Fluorescence Minus One (FMO) control. However, a low overall percentage of cells (29.38 ± 12.93%, mean ± SD) appeared in the positive gate. By 7 days post isolation, 44.94 ± 20.80% (mean ± SD) cells expressed tryptase were CD203c^+^, CD327/Siglec6^+^, with increased expression of FcεRIα, CD117, CD171/L1CAM, and MRGPRX2. The MC specific markers, FcεRIα, CD117, tryptase, CD327/Siglec6, and CD171/L1CAM increased during differentiation (w1 to w7), resulting in a highly pure population of MCs (86.59 ± 6.83% tryptase^+^). Although MRGPRX2 expression increased (w0 to w7) from 29.4%, peaking at 56.1% positive on average at week 7, the % of MRGPRX2 expressing cells was heterogeneous throughout, with a spread between donors from 8.7% to 87.4% (Figure 1A) at w9.

Despite the relatively stable and homogenous expression of other MC markers such as CD327/Siglec6, MRGPRX2 expression was bimodal, with both positive and negative cells within the mature cell population for each donor and ranged from low to relatively high percentage positivity (Figure 1B).

These data indicate that MRGPRX2 is low expressed at the initial stages of *in vitro* MC differentiation and increases thereafter. However, expression levels are heterogeneous and differ greatly between donors not only during differentiation but also in mature MCs.

### Substance P is a rapid and selective activator of MRGPRX2 in hMCs

**Figure 2:**
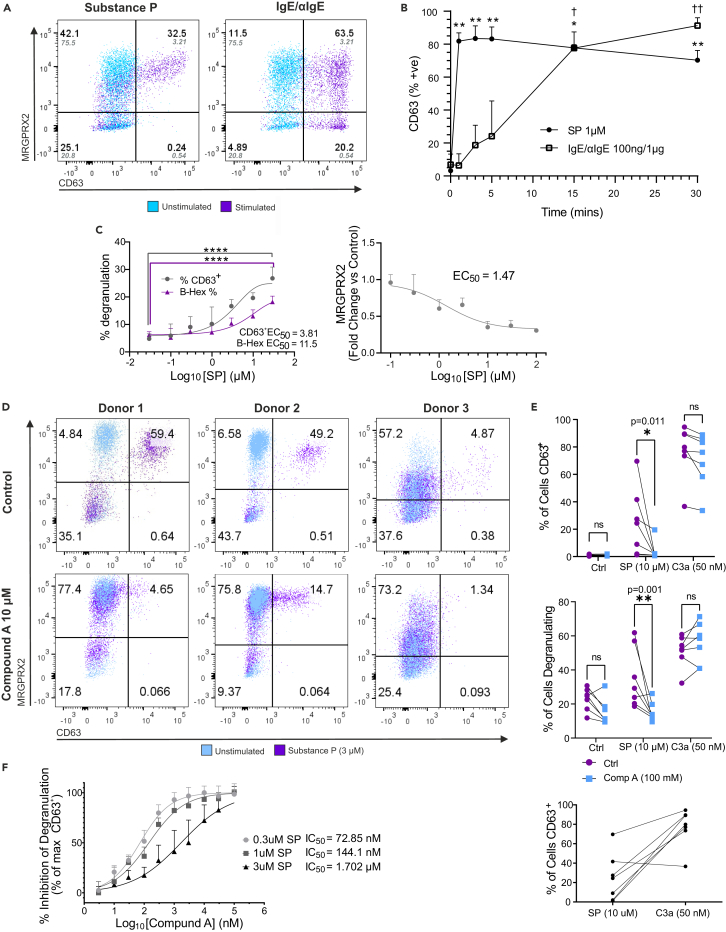
Substance P selectively activates MRGPRX2^+^ mast cells inducing rapid degranulation which is inhibited by compound A (A) Representative dot plots showing degranulation in mature MCs, as measured by CD63 externalization (x axis), vs. MRGPRX2 expression (y axis) after 15 min of treatment with Substance P (SP) (1 μM), or IgE/αIgE (100 ng/1 μg mL^−1^). Percentage of immuno-positive cells in each quadrant is shown for stimulated cells in black text. Unstimulated control percentages are shown in gray italics. (B) Time course of SP or IgE/αIgE induced degranulation (as indicated, 0–30 min), measured by flow cytometric analysis of CD63 externalization bound by CD63-specific fluorescent antibody. Data are mean ± SD of *n* = 3 from selected high MRGPRX2 expressing donors. Significant differences vs. time 0 are indicated (∗/† = *p* < 0.05, ∗∗/† † = *p* < 0.01), two-way ANOVA with Sidak’s multiple comparison post-test. (C) Substance P-induced degranulation as measured by CD63 externalization in conjunction with beta-hexosaminidase release (0.03–30 μM SP; left panel) and membrane expression of MRGPRX2 (0.1–100 μM SP; right panel) Data are mean ± SD of *n* = 3 replicates from mix of 3 donors *p* < 0.0001 two-way ANOVA with Dunnet’s multiple comparison post-test (left panel) or mean ± SEM of *n* = 3 from 3 donors (EC50 calculated by non-linear regression, three parameter best-fit curve; right panel). (D) Representative dot plots from three donors showing degranulation, as measured by flow cytometric analysis of immuno-specific CD63 externalization (x axis), vs. MRGPRX2 expression (y axis) after pretreatment with 10 μM compound A or vehicle control, for 30 min followed by 15 min of treatment with SP (3 μM), or vehicle control. Percentage of cells in each quadrant is shown for stimulated cells. Fluorescence Minus One (FMO) controls, with absence of specific antibody, for gating are shown in Figure S1. (E) Degranulation of MCs as measured by flow cytometric analysis of immuno-specific CD63 externalization (top panel) or beta-hexosaminidase enzyme release (middle panel) after pretreatment with 100 μM Compound A or vehicle control for 30 min, followed by stimulation with SP (10 μM), C3a (50 nM), or vehicle control for 30 min. Comparison between SP and C3a ligand induced degranulation on a per-donor basis is shown (lower panel). Data are *n* = 7 from 7 separate donors. *p* values as indicated (two-way ANOVA with Sidak’s multiple comparison post-test). (F) Inhibition of degranulation induced by 0.3, 1, or 3 μM SP after pretreatment with compound A (3 nM–100 μM). Data are mean ± SEM of *n* = 5 separate donors. Lines represent variable slope least squares fit, with calculated IC_50_ values labeled.

To investigate the dynamics and characteristics of MRGPRX2-induced MC degranulation, we stimulated hMCs with SP. For comparison we used the FcεRI engagement by IgE/αIgE complexes. As shown in Figure 2A, at 1 μM SP we observed that 43.6% of the MRGPRX2^+^ MCs were CD63^+^, and thus degranulated (Figures S1A, S1B, and 2A). In contrast, IgE/αIgE caused more than 80% of cells to degranulate. While SP induced degranulation only in MCs expressing MRGPRX2, MCs acquired membrane CD63 upon IgE/αIgE in an MGPRX2-independent manner (Figure 2A). Furthermore, MC degranulation upon SP stimulation was very rapid, resulting in >80% of CD63^+^ cells by 1 min post addition, decreasing slightly up to 30 min (Figure 2B). In contrast, IgE/αIgE-induced degranulation did not reach a significant level until 15 min post stimulation and peaked at 30 min (Figure 2B). Therefore, MRGPRX2 dependent degranulation is faster than IgE/αIgE. In a concentration-dependent curve, both CD63 externalization and β-hexosaminidase production reached a significant increase from the lowest concentration of SP (0.03 μM), even in cells with a lower level of MRGPRX2 (Figure 2C). Furthermore, MRGPRX2 surface expression decreased with increasing SP concentrations, with an EC50 of 1.47 μM (Figure 2C). When the reasons for this were investigated, no MRGPRX2 was shed into the media and there was no change in total MRGPRX2 detected in permeabilized cells (Figure S1C). Therefore, this finding is consistent with receptor internalization.

MCs were similarly responsive to a variety of other neuropeptides; vasoactive intestinal peptide (VIP), pituitary adenylate cyclase-activating peptide (PACAP), and pro-adrenomedullin peptide (PAMP) (Figure S2) although calcitonin gene-related peptide (CGRP) had minimal effect in inducing CD63 externalization. Significant degranulation was observed only in MRGPRX2 expressing cells in the case of PACAP, PAMP, and VIP (Figure S2A), within a concentration range similar to SP (0.01–100 μM) (Figure S2B).

These data show that neuropeptides selectively activate MRGPRX2 expressing MCs resulting in rapid MC degranulation.

### Substance P-induced degranulation is inhibited by compound A

We have shown that MC degranulation in response to SP depends on the presence of the MRGPRX2 receptor. To confirm this, we used compound A, a selective competitive inhibitor of MRGPRX2, and tested it on three donors with varying levels of MRGPRX2 expression. We applied an excess of MRGPRX2 inhibitor (10 μM) before exposing the cells to a sub maximal concentration of SP (3 μM). We observed degranulation only in those cells expressing MRGPRX2 (upper right quadrant, Figure 2D, control) and granules release was inhibited by preincubation of MCs for 30 min with compound A (upper right quadrant, Figure 2D, 10 μM compound A).

To demonstrate the selectivity of the inhibitor for MRGPRX2 dependent activities, we compared the inhibition of SP-induced degranulation, both in externalization of CD63 and β-hexosaminidase release, with that of another well characterized GPCR that causes degranulation, C3aR1.^3^ Both CD63 and β-hexosaminidase release induced by SP were significantly inhibited by compound A, despite the diversity of MC responses to SP itself (Figure 2E). In the same donors, compound A had no effect on degranulation induced by C3a acting on the GPCR C3aR1. Since there was no correlation between the response to SP and C3a in the same donors (Figure 2E, lower panel), we also demonstrated in the experiment that MRGPRX2 responsiveness variability was not due to a global cellular deficit in GPCR signaling (Figure 2E).

We further characterized compound A inhibition of SP-induced degranulation by pretreating cells with a concentration range of 3 nM–100 μM compound. This was followed by activation with 0.3, 1, and 3 μM SP. Compound A dose-dependently inhibited SP-induced degranulation in MCs. As a competitive antagonist of SP activation, the EC50 of the compound increased with increasing SP concentrations from 72.85 nM at 0.3 μM SP, to 144.1 nM at 1 μM SP, and to 1.7 μM at 3 μM SP (Figure 2F).

These data support the selectivity of MRGPRX2 for SP-dependent hMC degranulation.

### Substance P induced MC chemotaxis that is blocked by compound A

**Figure 3:**
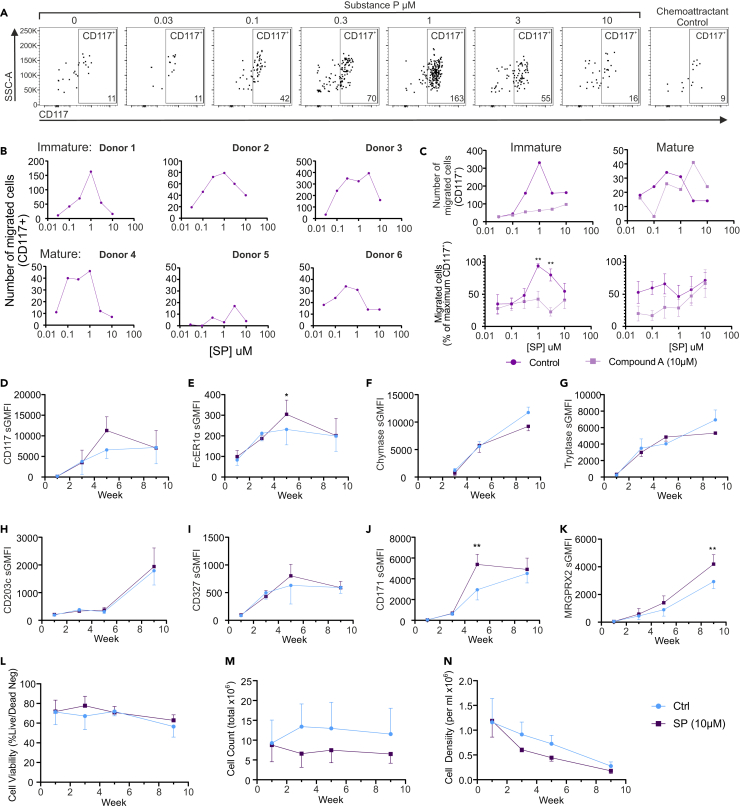
Substance P induced chemotaxis of immature and mature MCs and aids long term selection of MRGPRX2^+^ MC from progenitors (A) Representative dot plots showing migrated CD117+ immature MC recovered from the lower wells of the chemotaxis chamber after chemotaxis to 0–10 μM SP chemotaxis gradient, or equimolar (10 μM) SP on both sides (chemoattractant control). Cells were recovered and analyzed by counting the number of CD117-specific fluorescently labeled cells using flow cytometry (y axis) within the size parameter for MCp (SSC-A). The number of CD117+ cells is shown on the lower right of each plot. Plots show SSC-A (side scatter area) on the y axis and CD117 on the x axis. (B) Example of donor specific chemotaxis to SP in immature MC (donor 1–3) or mature MCs (donor 4–6). Number of migrating cells immuno-positive for CD117 (y axis) to each concentration of SP (x axis) as counted by flow cytometry is shown. (C) Inhibition of chemotaxis in immature (left) and mature (right) MCs in response to SP (0.05–10 μM [x axis]) in the presence of vehicle control (purple) or compound A (mauve). Upper panels show examples of single donor migration to SP. Lower panels are normalized mean data expressed as a percentage of the maximum number of migrated cells and are mean ± SEM of 6 (left) or 4 (right) experiments from separate donors. ∗∗ = *p* < 0.01 (two-way ANOVA with Sidak’s multiple comparison post-test). (D–N) Exposure of MCp to SP in cell culture medium during week 0–4 of differentiation. MCp were exposed to 10 μM SP added to media as cells were routinely cultured and media replenished. MC markers as labeled (D–K) and cell viability (L), total cell count (M), and cell density (N) are shown. Data are mean ± SEM of 3 donors. ∗ = *p* < 0.05, ∗∗ = *p* < 0.01 (two-way ANOVA with Sidak’s multiple comparison post-test).

Since MRGPRX2 is expressed on precursors of the MC lineage, and inflammation is diminished in the MRGPRb2 knock-out mouse,^15^ we hypothesized that MRGPRX2 could play a role in the recruitment of MCs from the blood into tissue during inflammation as previously demonstrated in mouse skin.^20^ Therefore, we established a model of MC chemotaxis using the ChemoTX chemotaxis chamber in immature (week 4–6) or mature (week 9–11) hMCs. We observed chemotaxis of CD117^+^ cells in response to SP, in donors that expressed high (>50%) MRGPRX2. Chemotaxis was not observed in the absence of chemoattractant (0) or in the presence of equal (10 μM) chemoattractant concentration on both sides of the chamber (chemoattractant control) (Figure 3A), indicating a specific chemotactic response. Individual donors of immature cells exhibited a higher number of migrating cells, and more uniform chemotaxis peaking at or around 1 μM SP. Mature donors migrated in lower numbers, and in a more variable concentration dependent pattern, with some donors migrating better at lower SP concentrations and others at higher concentrations (Figure 3B). Compound A inhibited both of these responses, and the pattern was again clear in immature MCs (Figure 3C). When aggregate data were normalized as a percentage of the maximum number of cells migrating from each donor (independent of the peak concentration), immature MCs migrated to SP with a peak at 1 μM SP that was significantly inhibited by compound A. Due to variability in the responses, inhibition of mature cell migration did not reach significance although the mean migration level was lower in the presence of compound A (Figure 3C).

To further validate the migration of hMCs to SP, we attempted to visualize migration on an Incucyte plate imager over 6 h. In fibronectin coated wells, we observed MCs moving toward the central pores of the plate, although cells did not migrate through the pores (Video S1). Similar to the data obtained from the ChemoTX plate, the number of cells migrating into the center of the plate peaked around 1–10 μM SP, showing a clear increase from control (Figure S3A). We carried out a migration assay to observe MC migration around a fibronectin coated flat bottomed plate over 6 h using Incucyte imaging. Manual tracking of individual cells revealed a peak of 10.95 ± 1.28 (mean ± SD) μm.min^−1^ at 0.1 μM SP. Compound A inhibited this migration in terms of both movement velocity and distance (Figure S3B). While compound A inhibited SP induced migration, aprepitant, a selective neurokinin 1 receptor antagonist, did not (Figure S3D), confirming the MRGPRX2 dependent activation of SP.

Video S1. Video of mast cells migrating from the outside toward the central pores of a migration plate, related to Figure 3 Video was captured on an Incucyte S3 Live Cell Analysis system and 8 μm pore ClearView migration plate capturing images at 20 min intervals over a total of 6 h. Cells migrate toward central pores of migration plate.

Taken together, these data show that SP-induced chemotaxis of MCs is MRGPRX2 dependent and might be key in MC progenitor recruitment to or within tissues.

### Long-term supplementation of MC culture with SP aids selection of MRGPRX2 expressing cells

SP expressing neurons are present in barrier tissues where MCs are commonly found. Therefore, MCs located proximal to such neurons might be subjected to SP for extended periods particularly during neurogenic inflammation. Therefore, we exposed MC progenitors to 10 μM SP containing medium, or vehicle control, continually during the first 4 weeks of culture. SP-exposed cells exhibited significantly higher FcεR1a and CD171/L1CAM expression at week 5 of differentiation (Figures 3E and 3J) but not in mature cells, while the overall cell number, but not viability, was lower (Figures 3L–3N). MRGPRX2 specific mean fluorescence was significantly higher in mature MCs (Figure 3K), which appeared to be distinct from control cells early in differentiation. These data indicate that the presence of SP in culture media during MC differentiation leads to early selection of MC precursors with greater expression of MC specific markers. This gives rise to cells expressing greater MRGPRX2 levels.

### MRGPRX2^+^ MCs are increased in inflammatory skin conditions and locate in proximity of SP^+^ nerve fibers during skin reinnervation *in vitro*

To investigate whether MRGPRX2^+^ MC numbers correlate with inflammatory conditions and thus indicate that MRGPRX2 is a marker of MC accumulation in the tissue, we have investigated MCs in psoriasis skin lesions and compared them to healthy skin.

**Figure 4:**
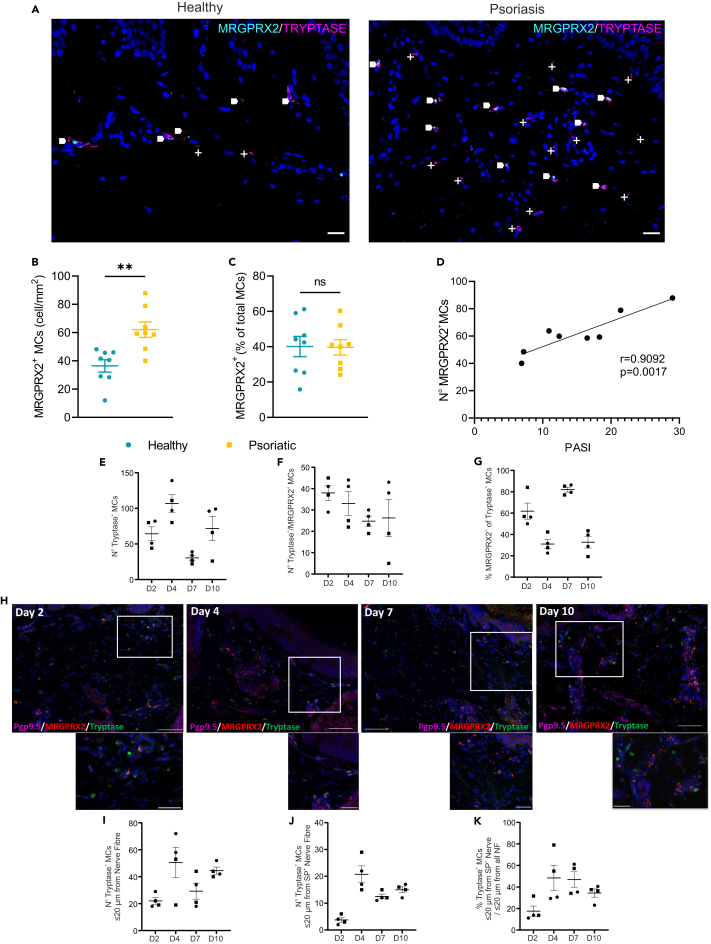
MRGPRX2^+^ mast cells are increased in inflammatory skin conditions and locate in proximity of SP + nerve fibers (NFs) during skin reinnervation *in vitro* (A) Representative images of healthy and psoriasis skin sections taken from skin punch biopsies, stained with anti-sera to MRGPRX2 (cyan) and MC tryptase (magenta) with DAPI (blue) nuclear counterstain. Areas of colocalization of MRGPRX2 and tryptase are indicated by arrows. MRGPRX2 negative MC are marked by +. Scale bar: 20 μm. (B and C) Density (B) and percentage (C) of MRGPRX2+ MCs in healthy and psoriasis skin sections. Cell numbers were obtained by manual counting of images as described in the STAR Methods section. Data are mean ± SEM of *n* = 6 donors. ns = not significant (unpaired t test). (D) Correlation between psoriasis area and severity index (PASI) and MRGPRX2+ MC density in psoriasis skin sections, measured by Pearson’s correlation coefficient. (E–G) Number of (N°) tryptase^+^ MCs (E), tryptase^+^/MRGPRX2^+^ MCs (F), and percentage of (%) tryptase positive MCs (G) which are MRGPRX2^+^ during and after the course of 10 days of culture for skin reinnervation/nerve sprouting. Cell numbers were obtained by manual counting of images as described in the STAR Methods section. Of note: all the layers of the skin are reinnervated by day 5. Data are mean ± SEM of *n* = 4 sections (dermis and epidermis on each section were imaged in whole at 200× and stitched for analysis) from *n* = 2 biopsies from 1 donor. (H) Representative images of human skin biopsies after 2, 4, 7, and 10 days of co-culture with sensory neurons and stained with anti-sera to PGP9.5, tryptase and MRGPRX2 and counterstained with DAPI nuclear stain. Scale bar: 50 (high magnification insert) or 100 μm. (I–K) Number and percentage of tryptase^+^ MCs in proximity to PGP9.5^+^ or SP^+^ NFs (within 20 μm from NFs) during and after the course of skin culture reinnervation/nerve sprouting. Of note: all the layers of the skin are reinnervated by day 5. Data are mean ± SEM of *n* = 4 sections from *n* = 2 biopsies from 1 donor.

The association between MRGPRX2^+^ MCs and NF markers in skin from psoriasis lesions and healthy volunteers was investigated. As previously described^22^ and shown in Figures 4A and S4B, the number of MCs in sections from psoriasis lesions was increased compared to normal skin. Furthermore, the frequency of MRGPRX2^+^ MCs was significantly higher in psoriasis lesions compared to normal skin MCs (Figure 4B). Notably, the expression of MRGPRX2 in skin MCs was also heterogeneous, and the percentage of MCs expressing MRGPRX2 (healthy 45.40 ± 16.30%, psoriasis 39.65 ± 12.29%; Figure 4C) was similar to that of mature blood derived MCs (46.82 ± 28.66%; Figure 1A). The density of MRGPRX2^+^ MCs was significantly correlated with psoriasis severity (Figure 4D), although the percentage of MRGPRX2^+^ MCs was not increased in psoriasis (Figure 4C) indicating that changes in MRGPRX2 density are likely due to changes in overall MC numbers as we have previously demonstrated.^22^ Altogether these data indicate that MRGPRX2^+^ MCs accumulate in inflamed skin and their number correlates with the severity of the disease.

To investigate whether MRGPRX2^+^ MCs tissue locate preferentially in proximity of NFs and whether they are actively recruited via SP released during NF sprouting, we used a human skin organ culture in which reinnervation was induced via the addition of human sensory neurons (differentiated from neural progenitors cells) and co-culture was performed for 10 days.^23^

We wanted to understand next if MRGPRX2^+^ MCs were migrating in close proximity to SP^+^ NFs preferentially, and if their number was modulated during reinnervation/nerve sprouting. To do so, we have used a previously published fully humanized reinnervated skin organ culture model^23^ and evaluated MRGPRX2^+^ MC (tryptase^+^) numbers and their proximity to NFs (PGP9.5^+^), at different time points during the co-culture, by immunofluorescence. We look at the number of MCs and their proximity with PGP9.5^+^ NFs during the course of reinnervation/nerve sprouting (from 0 to 5 days of co-culture) and after the completion of reinnervation/nerve sprouting (after 5 days of co-culture, as we previously showed that reinnervation is complete after 5 days of co-culture by the presence of PGP9.5 NFs in the different layers of the epidermis.^23^ Using this experimental set-up, we aim to understand if MCs are attracted by NFs before or after the completion of the reinnervation and reconnection of the different structures (dermis, epidermis, hair follicles, and sweat glands) with NFs.

Our data showed that the number of MCs (tryptase^+^) in the papillary dermis increased between day 2 and day 4; days in which the full reinnervation of skin is not yet complete, while this number decreased at day 7 (just after the completion of reinnervation). It increased again at day 10 (Figure 4E). While the number of tryptase^+^/MRGPRX2^+^ MCs was at the highest at day 2 slightly decreasing thereafter, the % of tryptase^+^/MRGPRX2^+^ MCs among the total number of tryptase^+^ cells was at lowest levels at day 4 and day 10 (Figures 4F and 4G). Furthermore, the highest number of tryptase^+^ MCs found in the proximity of SP^+^ NFs was at day 4 (Figure 4I), coinciding with the highest number of tryptase^+^ cells in the papillary dermis. Moreover, these MCs were preferentially associated with SP^+^ NFs (over the total number of PGP9.5^+^ NFs) from day 4 to day 7 (days just before and after complete reinnervation) (Figures 4J, 4K, and S4C).

In summary, these results indicate that MCs during sensory nerve reinnervation/sprouting preferentially associate with SP^+^ fibers and that a high numbers of MRGPRX2^+^ MCs are present in close proximity with NFs (SP^+^ preferentially) very early during the course of reinnervation/nerve sprouting indicating SP-mediated recruitment and a role of MRGPRX2/SP^+^ NFs communication in skin physiology, likely reflecting the natural innervation state of the skin.

## Discussion

In the present study, we investigated the significance of MRGPRX2 in controlling the activation and degranulation of hMCs, highlighting its role in inflammation. Our findings reveal a notable characteristic of MRGPRX2, namely its heterogeneous expression among individuals. This heterogeneity persists through the processes of cell differentiation, maturation, and under inflammatory conditions. A key contribution of our study is the identification of an activity within the MRGPRX2-SP signaling pathway. We demonstrated that at high concentrations, the tachykinin SP induces MC degranulation, while at low concentrations, SP modulates the chemotaxis of MC progenitors. Importantly, both of these activities are contingent upon SP binding to MRGPRX2.

Prior studies have revealed varied MRGPRX2 expression in MCs from diverse anatomical sites. Skin-derived MCs exhibit the highest expression under normal conditions, while lung MCs appear to lack expression.^5^^,^^7^^,^^24^^,^^25^ Based on early studies, SP levels have been found to be higher in the lungs, bronchoalveolar lavage, and sputum of asthmatic patients compared to normal controls.^26^^,^^27^ However, the expression of MRGPRX2 on human lung MC is a topic of debate. This controversy may stem from the methods used for detection; while immunofluorescence can identify cytoplasmic MRGPRX2, flow cytometry has not provided evidence of MRGPRX2 receptor expression in human lung cells.^7^^,^^18^^,^^28^^,^^29^ Furthermore, SP does not induce bronchoconstriction in asthmatic subjects^30^ suggesting a lack of direct effect on MCs. As a result, it remains to be proven whether the SP-MRGPRX2 pathway regulates MC chemotactic activities in the lung.

Our investigation demonstrates uniform increases in MC markers (FcεRI, Siglec6, and L1CAM) during differentiation. However, both progenitors and mature MCs display high heterogeneity in MRGPRX2 expression levels and percentages among individuals. The role of MRGPRX2 variants, as suggested by Alkanfari et al.,^31^ remains unclear, as do the key modulators and their potential tissue specificity.

That SP plays a role in the maintenance of MRGPRX2 is demonstrated in our study by the observation that in the presence of SP in the culture medium, a higher number of MCs express MRGPRX2.

In psoriasis, we observed an increase in MRGPRX2 expression, likely linked to elevated MC numbers in the inflammatory skin disease. This suggests that when expressed, MRGPRX2 is maintained at constitutive levels, differing from the upregulation driven by another tachykinin, hemokinin-1 in asthmatic lung cells.^18^

MRGPRX2 is known to bind a variety of ligands. In our study, we have confirmed that some neuropeptides selectively activate MRGPRX2 (and not NK1R) expressing MCs in a physiological concentration range that results in rapid MC degranulation. This confirms previous findings indicating the differences in the time of response between SP and FcεRI-mediated degranulation.^32^

Al Hamwi et al.^33^ recently revealed CXCL14 as a ligand that activates MRGPRX2, expanding its ligand repertoire. This emphasizes MRGPRX2 as a receptor capable of modulating MC chemotaxis in diseases. Our data align with this concept. While CXCL14-mediated MC activation and chemotaxis may contribute to idiopathic lung fibrosis with an excess of the chemokine, our findings suggest that the SP-MRGPRX2 pathway plays a crucial role in inflammatory skin conditions, neurogenic inflammation, including itch, psoriasis, and urticaria.^24^^,^^34^^,^^35^^,^^36^

Various chemokines (e.g., CXCL10), cytokines (e.g., stem cell factor [SCF]), and lipid mediators (e.g., PGE2 and LTB4) have been implicated in an autocrine/paracrine MC migration.^37^^,^^38^^,^^39^^,^^40^^,^^41^ While LTB4 is known to attract MC progenitors, recruiting them from the circulation, and lose its effect upon MC maturation,^38^^,^^40^^,^^42^ PGE2 is more prone to regulating cell movement within the tissue. Progenitor-derived MCs have previously been shown to exhibit biphasic responses, migrating in an S1P-PI3K dependent, G-protein independent manner at low concentrations of antigen, while degranulating at higher antigen concentrations.^43^ It is unknown whether MRGPRX2 can signal in this way, although other aspects of MRGPRX2 and FcεRIα signaling appear to be shared.^44^

Interestingly, both LTB4 and PGE2 are secreted upon MC stimulation by SP.^39^^,^^45^^,^^46^ In our study, we demonstrated that SP chemotactic activity mainly targets progenitor cells and occurs at low concentrations. It is likely that the propensity to degranulate increases both with cell maturity and proximity to the stimulus and so physiologically MCs could avoid degranulation during migration toward a stimulus while degranulating at the point of inflammation where concentration is maximal. Variability in the migratory response to SP in mature cells seems to result from an interplay between donor dependent factors including level of MRGPRX2 expression on individual cells and the number of cells in the population expressing the receptor. Similar donor variability has been shown in responsiveness to PGE2.^41^ Whether MC migration is a direct autocrine or paracrine effect of SP, or whether this is indirect and mediated via LTB4, is not yet clear and needs further investigation. The role of SP and LTB4 may also be important in MC-NF communication^47^^,^^48^^,^^49^^,^^50^ such as in itch.^51^

In summary, we provide a new role for the SP-MRGPRX2 pathway in controlling MC repositioning in the tissue and its implications in neurogenic inflammatory conditions as well in tissue homeostasis and NF development and repair.

### Limitations of the study

While the study sheds light on new MRGPRX2-driven activities in hMCs, our research remains an *in vitro* study that requires validation in skin *ex vivo* models of inflammation. Additionally, it is essential to clarify whether SP-induced MC migration is a property shared by other neuropeptides, particularly CGRP that we have previously shown to be associated with psoriasis severity.^22^ Our blood donors were fully anonymized and so the association of study outcomes with sex characteristics for hMCs is not discernible, although the large number of donors used and the national balance in donor sex (44% male) would indicate broad generalizability of our results.

## Resource availability

### Lead contact

Further information and requests for resources and reagents should be directed to and will be fulfilled by the lead contact, Silvia Bulfone-Paus (silvia.bulfone-paus@manchester.ac.uk).

### Materials availability

This study did not generate new unique reagents.

### Data and code availability

• Source data will be shared by the lead contact upon request.
• This paper does not report original code.
• Any additional information required to reanalyze the data reported in this paper is available from the lead contact upon request.

## Acknowledgments

The authors wish to thank Dr Chiara Tontini for assistance in preparing the graphical abstract. The Bioimaging Facility microscopes used in this study were purchased with grants from 10.13039/501100000268BBSRC, 10.13039/100004440Wellcome, and the University of Manchester Strategic Fund. Special thanks go to the bioimaging staff for their help with microscopy. The Flow Cytometry Facility equipment used in this study was supported by funding from the Manchester Collaborative Centre for Inflammation Research, Wellcome Trust, and University of Manchester Strategic Fund. The research and P.W.W. were supported by a GSK research grant. S.B.-P. and R.B. are funded by MRC (MR/S036954/1). J.C. was supported by a grant from CUTANEON.

## Author contributions

P.W.W., J.C., R.B., C.H.M., and S.B.-P.: conceptualization; P.W.W., J.C., R.B., and O.K.: investigation and methodology. Z.W. and C.H.M.: provided resources; P.W.W., J.C., R.B., O.K., and S.B.-P.: formal analysis; P.W.W., S.B.-P.: funding acquisition and supervision; P.W.W., J.C., R.B., and S.B.-P.: writing – original draft, and all authors: writing – review & editing. All authors have read and agreed to the published version of the manuscript.

## Declaration of interests

Z.W. and C.H.M. are employees at GSK Collegeville.

## STAR★Methods

### Key resources table

**Table:** 

REAGENT or RESOURCE	SOURCE	IDENTIFIER
**Antibodies**		

Goat Anti-Human IgE (Epsilon Chain)	LGC SeraCare - KPL	Cat#5210-0158; RRID: AB_2773725
Goat Anti-Mouse IgG PE	Biolegend	Cat#405307; RRID: AB_315010
Goat Anti-Mouse IgG APC	Biolegend	Cat#405308; RRID: AB_315011
Mouse Anti-Human CD117	Biolegend	Cat#313230; RRID: AB_2566217
Mouse Anti-Human CD203c	Biolegend	Cat#324606; RRID: AB_756043
Mouse Anti-Human CD327/Siglec6	Biotechne	Cat#FAB2859T
Mouse Anti-Human CD45	BD Bioscience	Cat#563792; RRID: AB_2744400
Mouse Anti-Human CD63	Biolegend	Cat#353012; RRID: AB_10915273
Mouse Anti-Human FcεRIα	Biolegend	Cat#334628; RRID: AB_2566505
Mouse Anti-Human MRGPRX2	Biolegend	Cat#359008; RRID: AB_2783262
Mouse Anti-Human MRGPRX2	Biolegend	Cat#359004; RRID: AB_2562301
Mouse Anti-Human Mast Cell Tryptase (clone AA1)	Biolegend	Cat#369402; RRID: AB_2566541
Mouse Anti-Human Chymase	Merck Millipore	Cat#MAB1254; RRID: AB_2083625
Lineage Cocktail, FITC	ThermoFisher Scientific	Cat#22-7778-72; RRID: AB_1311229
Chicken anti-Rabbit IgG H+L (Alexa Fluor™ 647)	ThermoFisher Scientific	A21443; RRID: AB_1500685
Goat anti-Guinea Pig IgG H+L (Alexa Fluor™ 594)	ThermoFisher Scientific	A11076; RRID: AB_2534120
Goat anti-Rabbit IgG H+L (Alexa Fluor™ 488)	ThermoFisher Scientific	A11034; RRID: AB_2576217
Goat anti-Rabbit IgG H+L (Alexa Fluor™ 555)	ThermoFisher Scientific	A21428; RRID: AB_2535849
Mouse Anti-MRGPRX2	Origene	Cat#AM26724 Clone 1D3
Rabbit anti-PGP9.5	AbCam	ab108986; RRID: AB_10891773
Guinea Pig Anti-Substance P	AbCam	ab106291; RRID: AB_10864733
Mouse Anti-Mast Cell Tryptase (clone AA1)	AbCam	ab2378; RRID: AB_303023
VectaFluro Duet Kit	Vector Labs	DK8818

**Biological samples**		

Anonymized Human Leukocyte Cones from healthy donors	NHS Blood and Transplant, UK	
Human Skin Biopsies	Salford Royal NHS Trust, UK	
Human Scalp Skin	University of Miami,Miller School Of Medicine, USA	

**Chemicals, peptides, and recombinant proteins**		

Compound A	Macphee, C.H et al.	submitted
Substance P (SP)	Genscript	Cat#RP10178
Aprepitant	Sigma-Aldrich	SML2215
Calcitonin Gene Related Peptide (CGRP)	Sigma-Aldrich	Cat#C0167
Vasoactive Intestinal Peptide (VIP)	Sigma-Aldrich	Cat#V6130
Pro-Adrenomedullin Peptide 9-20 (PAMP 9-20)	Genscript	Custom Synthesis Lot#U6169FF250-1/PE7757
Pituitary Adenylate Cyclase-Activating Peptide 1-27 (PACAP 1-27)	Tocris	Cat#1183
Pituitary Adenylate Cyclase-Activating Peptide 1-38 (PACAP 1-38)	Tocris	Cat#1186
C3a	Complement Technology	Cat#A118
4-Nitrophenyl N-acetyl-β-D-glucosaminide	Sigma Aldrich	Cat#N9376
Recombinant Stem Cell Factor (SCF)	Genscript	Cat#Z02692-1
Recombinant IL-6	Genscript	Cat#Z03034-1
Recombinant IL-3	Genscript	Cat#Z03156-1
Recombinant Nerve Growth Factor	Sigma Aldrich	Cat# N1408
Recombinant Human IgE, Myeloma	Sigma Aldrich	Cat#401152
Calcein Green AM	ThermoFisher Scientific	Cat#34852
Live/Dead™ Fixable Blue Dead Cell Stain	ThermoFisher Scientific	Cat#L34962

**Critical commercial assays**		

CD117 Microbead kit, human	Miltenyi Biotec	Cat#130-091-332
ChemoTX Chemotaxis Plates	Neuroprobe	Cat#116-8
Incucyte Clearview Migration Plate	Essen Bioscience	Cat#4582

**Software and algorithms**		

Image J	NIH	v1.53g
Prism	GraphPad	v9.1.2,
Chemotaxis and Migration Tool	Ibidi	v2
FlowJo	BD	v10.10.0

**Other**		

StemSpan SFEM	Stemcell Technologies	Cat#09650

### Experimental model and study participant details

This manuscript reports results from human subjects as reported below.

#### Human skin and blood samples

Six-millimeter skin punch biopsies from psoriatic and healthy subjects were obtained from Salford Royal Hospital, following protocols approved by the local NHS research ethics committee (13/NW/0867 & 10/H1005/77). Peripheral blood NC24 leukocyte cones from *n* = 38 anonymous healthy donors were obtained from NHS Blood and Transplant (Manchester, UK) under a material transfer agreement and used in accordance with a protocol approved by the University of Manchester research ethics committee (UREC ref: 2018-2696-5711). All subjects gave informed consent. Demographic data for skin samples is shown in Table S1.

#### Human scalp skin

Temporal and occipital human scalp skin was obtained from a 25 year-old healthy female Caucasian donor undergoing routine face-lift surgery in the United States. The use of these human discarded tissues is considered non-human subject research and exempted under 45 CFR46.101.2 by the Institutional Review Board of the University of Miami, Miller School of Medicine (Miami, FL). Human scalp samples were obtained 1 day after surgery, and 4 mm skin biopsies were performed and used for *in vitro* skin re-innervation^23^^,^^50^ as described below.

#### Human Mast Cells

Peripheral blood-derived human mast cells were generated from peripheral blood leukocyte cones.^3^ PBMCs were obtained via layering blood over a Ficoll (1.077g/mL) density gradient followed by centrifugation. CD117^+^ progenitor cells were isolated by positive magnetic selection using the CD117^+^ human microbead kit according to the manufacturer’s instructions (Miltenyi Biotec, Bisley, UK). Day 0 cells are regarded as MCp in this manuscript. Cells were grown in media containing human IL-3, human IL-6, and human stem cell factor for 28 days, after which the medium was changed, and IL-3 was withdrawn. These cells were regarded as immature MC. Cells were tested for maturity at 8-10 weeks by flow cytometric analysis of CD117 and FcεRI expression and degranulation in response to IgE/αIgE. At this point cells are regarded as mature MCs.

#### MRGPRX2 inhibitor

The MRGPRX2 inhibitor, Compound A, was manufactured and supplied by GSK (Collegeville, USA). The pharmacology of the inhibitor is the subject of a separate publication (manuscript in preparation). Compound A was reconstituted in DMSO and applied 30 min prior to experiments, to a maximum final DMSO concentration of 0.1% (v/v) in the cell culture medium.

### Method details

#### Flow cytometric analysis

For analysis of differentiation, samples of cultured cells were analysed immediately after isolation, and after 1, 3, 5, 7 and 9 weeks of differentiation as above. Cells were washed in FACS buffer (PBS,2% FBS, 200 μM EDTA) and incubated with Fc receptor blocking reagent prior to the addition of antibodies specific to FcεRIα (clone AER-37), CD117(clone 104D2), CD203c (clone NP4D6), CD123 (clone 6H6), CD171/L1CAM (clone L1-OV198.5), MRGPRX2 (clone K125H4), (all from Biolegend, London, UK), CD45 (clone HI30), haematopoietic lineage cocktail (clones RPA-2.10, OKT3, 61D3, CB16, HIB19, TULY56, HIR2) (ThermoFisher Scientific, Paisley, UK) and CD327/Siglec6 (clone 767329, R&D systems, Abbingdon, UK). Degranulation was assessed by labelling of externalised CD63 (clone H5C6) as previously established^3^ in response to substance P (Genscript, Oxford, UK), IgE/anti-IgE, anti-FcεRIα (clone AER37, Biolegend, London, UK) or C3a (CompTech, Tyler, USA). In all cases, cells were washed in PBS prior to incubation with live/dead™ reagent before analysis. For intracellular detection of tryptase (clone AA1, Biolegend, London, UK) and chymase (clone B7, Merck Millipore, Watford, UK), cells were washed and incubated with live/dead™ reagent prior to fixation with 4% formaldehyde solution. Fc blocking reagent and antibodies were added, diluted in 1x permeabilisation buffer (BD Bioscience, Winnersh, UK), cells were washed and incubated with goat anti-mouse polyclonal antibodies labelled with PE or APC (Biolegend, London, UK). All cells were analysed on FACSymphony A5 or LSRFostessa instruments, and FlowJo software (BD Bioscience, Winnersh, UK). An example of the gating strategy used for MCs is shown in Figure S1. CD63 externalisation was used as a surrogate for MC degranulation according to established methodology.^52^^,^^53^^,^^54^

#### Beta-hexosamindiase assay

Beta-hexosaminidase release was quantified in a ratiometric manner by comparison of equal amounts of cell pellet and supernatant. Cell pellets were harvested by centrifugation and supernatant removed. Cells were lysed in 1% (v/v) Triton-X100 diluted in fresh cell culture medium. Colorimetric β-hexosaminidase substrate (2.5 mM 4-nitrophenyl N-acetyl-β-D-glucosamine in 50 mM citrate buffer (pH4.5)) was added for 2 hours at 37°C before the reaction was stopped by the addition of 0.2 M glycine (pH10). OD was measured at 405 nm on an Inifinite M200 Pro plate reader (Tecan, Reading, UK). Degranulation was assessed as the percentage released expressed as a percentage of the total β-hexosaminidase.

#### Chemotaxis and migration assays

Chemotaxis assays were carried out using 8 μm pore ChemoTX™ assay plates (Neuroprobe, Gaithersburg, USA). Cells were first loaded with 5 μM Calcein-AM for 30 mins, 37°C/5% CO_2_ in cell culture medium prior to being washed and resuspended at a concentration of 1 x 10^6^.ml^-1^. Where appropriate cells were pre-incubated with Compound A or vehicle control. Chemoattractants were prepared in cell culture medium and placed in the lower chamber. The plate was constructed and 50 μl cell suspension applied to the upper part of the membrane of each well. No chemoattractant and equal chemoattractant on both sides of the chamber were used to control for random cell movement. The plate was incubated for 6 hours 37°C/5% CO_2_ before analysis of migrated cells, as follows. Cells were removed from above the membrane by pipetting, the membrane was then washed with PBS buffer containing 2mM EDTA and wiped using a rubber filter wiper (Neuroprobe). PBS/EDTA was applied to the upper side of the membrane and the plate incubated for 30 mins at 4°C before again being wiped. The plate was then centrifuged at 500 x *g* for 5 mins to capture cells attached to the underside of the membrane. Non-adherent migrated cells were then removed from the plate, any remaining adherent cells removed by incubation in ice cold FACS buffer for 5 mins and then combined with non-adherent cells. Total migrated cells were subject to flow cytometric analysis for cell surface expression of FcεRIα, CD117 and MRGPRX2 as described above. Total migrated cells were counted as Live/Dead™-/Calcein-AM+/CD117+ cells or Live/Dead™-/Calcein-AM+ cells in each sample.

#### Immunofluorescence staining of psoriasis skin samples

Five-micrometer paraffin-embedded skin sections were de-paraffinized in xylene and rehydrated through graded alcohols. Slides were boiled in antigen retrieval buffer (10 mM Citrate buffer, pH 6) for 20 mins and allowed to cool. Non-specific binding was blocked using 5% normal horse serum diluted in PBS-Tween 20 (0.1% v/v) for 30 mins. Sections were subsequently incubated with primary antibodies specific to tryptase (clone: EPR9522, 1:4000, Abcam, Cambridge, UK), and MRGPRX2 (clone 1D3, 1:100, Origene, Rockville, USA) diluted in blocking buffer for an hour at room temperature. The VectaFluor™ Duet Kit (DyLight 488 anti-rabbit IgG & DyLight 594 anti-mouse IgG; DK8818, Vector Labs) was used for visualization for 30 mins at room temperature. Sections were mounted using Fluoroshield mountant containing 4',6-diamidino-2-phenylindole (DAPI) before image analysis on an Olympus BX51 upright fluorescent microscope equipped with bandpass filters for DAPI, FITC, and TexasRed. Images were analyzed using Image J software (https://imagej.nih.gov/ij/download.html).

#### *In vitro* re-innervation skin model

##### nSCs In vitro culture

Human induced pluripotent stem cell-derived nSCs (Ax0015, Axol Bioscience, Cambridge, United Kingdom) were differentiated in a mixture of DMEM (Gibco, 11965092) +DMEM/Ham’s F12 (Gibco, 11320033) (3:1) supplemented with 25 ng/ml of nerve growth factor-beta (β-NGF) (Sigma-Aldrich, St. Louis, MO, N1408) and plated onto 0.1% gelatin (Sigma-Aldrich, S2500) -coated wells at a density of 15,000 cells per well in a 12-well plate for 5 days before adding scalp skin punches.

##### nSC coculture with human skin

Our study used 4 mm skin punches (2 punches per experimental group) randomly assigned to each experimental study condition and placed on top of a layer of 15,000 human induced pluripotent stem cell-derived nSCs (Ax0015) per well before being cultured at 37°C with 5% carbon dioxide in a mixture of DMEM (Gibco, 11965092) +DMEM/Ham’s F12 (Gibco, 11320033) (3:1) supplemented with 25 ng/ml β-NGF (Sigma-Aldrich, N1408), 2 mM of L-glutamine (Gibco, 25030149), 10 ng/ml hydrocortisone (Sigma-Aldrich, H0135), 10 μg/ml insulin (Sigma-Aldrich, I9278), and 1% penicillin/streptomycin mix (Gibco, 15140122). The culture medium was replaced every other day. After 2, 4, 7, and 10 days of coculturing, punches were fixed for 3 hours in 4% paraformaldehyde (Sigma-Aldrich, 1.00496.5000) and overnight in 10% sucrose (Sigma-Aldrich, S7903) before embedding into cryomatrix (Epredia, 6769006) and snap-freezing in liquid nitrogen. The tissue blocks were sectioned in a cryostat (Cryostar NX50, Thermo Fisher Scientific) at 25 μm (for nerve stainings).

##### PGP9.5/Substance P/Tryptase staining

The already fixed cryosections were washed three times for 5 minutes using Phosphate Buffer Saline (PBS) and preincubated in 10% Bovine Serum Albumin (BSA) (Sigma-Aldrich, A9418) for 30 min at RT. Primary antibodies for PGP9.5 (Abcam, ab108986; 1/500) and substance P (Abcam, ab106291; 1/250) were diluted in 2% BSA, 0.3% Triton X-100 (Sigma-Aldrich, X100) in PBS and incubated overnight at 4°C, followed by a secondary Alexa Fluor 647 and 594 respectively (Life Technologies, A21443 and A11076 respectively) antibody diluted in 2% BSA, 0.3% Triton X-100 in PBS for 1 hr at RT. After blocking the sections with 0.5% Triton-X100 + 10% goat normal serum (VWR, 103219-584) in PBS for 30 mins at RT, the primary antibody for tryptase (Abcam, ab2378) was diluted in 0.3% Triton X-100 in PBS and incubated overnight at 4°C, followed by a secondary antibody Alexa Fluor 488 (Life Technologies, A11034) in PBS for 1 hr at RT. The cryosections were counterstained and mounted with DAPI/Fluoromount-G (Electron Microscopy Sciences, 17984-24).

##### PGP9.5/MRGPRX2/tryptase staining

The already fixed cryosections were washed three times for 5 mins using Phosphate Buffer Saline (PBS) and preincubated in 10% Bovine Serum Albumin (BSA) (Sigma-Aldrich, A9418-50G) for 30 mins at RT. Primary antibodies for PGP9.5 (Abcam, ab108986; 1/500) and MRGPRX2 (Origene, AM26724; 1/200) were diluted in 2% BSA, 0.3% of Triton X-100 (Sigma-Aldrich, X100) in PBS and incubated overnight at 4°C followed by a secondary Alexa Fluor 647 and 555 respectively (Life Technologies, A21443 and A21428 respectively) antibody in diluted in 2% BSA, 0.3% of Triton X-100 in PBS for 1hr at RT. After blocking the sections with 0.5% Triton-X100 + 10% goat normal serum (VWR, 103219-584) in PBS for 30 mins at RT, the primary antibody for tryptase (Abcam, ab2378; was diluted in 0.3% of Triton X-100 in PBS and incubated overnight at 4°C followed by a secondary antibody Alexa Fluor 488 (Life Technologies, A11034) in PBS for 1 hour at RT. The cryosections were counterstained and mounted with DAPI/Fluoromount-G (Electron Microscopy Sciences, 17984-24). All the pictures were taken using our Biozero BZ-X700 (Keyence, Japan) All-in-one Fluorescent microscope and full focus adjustments were proceed with the BZ analyzer (Keyence, Japan).

##### Nerve fibers quantitative immunohistomorphometry

Immunofluorescent staining for PGP9.5 or SP were performed on 25-μm-thick sections to detect intra-dermal NFs and our number of sections corresponds to the guidelines of the Peripheral Nerve Society.^55^ We used the full focus adjustment from our BZ-analyzer (Keyence, Japan) to count all the distinct immunoreactive fibres. For intra-dermal NFs, we used the corresponding Dermo-epidermal junction (DEJ) line that we transfer at the level of the border between the papillary and reticular dermis.^23^ The whole dermis and epidermis were imaged at 200x magnification and individual optical fields were stitched to visualize the whole section to better follow each NF and avoid duplications.

##### Quantitative immunohistomorphometry analysis of MRGPRX2+ and Tryptase+ cells

For quantitative (immuno)-histomorphometry MRGPRX2 and Tryptase-positive cells were counted in the papillary dermis (200um from the DEJ), and the total number and the percentage of positive cells were determined. For the number of MRGPRX2 and Trypatse-positive cells in contact with NFs, only positive cells within 20um from NFs were considered. The whole dermis and epidermis were imaged at 200x magnification and individual optical fields were stitched to visualize the whole section to better follow each NF and properly evaluate the proximity of each positive cell with each NF. This allows to avoid duplications.

### Quantification and statistical analysis

Graphing and analysis was carried out in Prism (v9.1.2, GraphPad, SanDiego, USA). Data were subject to normality testing (Kolmogorov-Smirnov test) are presented as mean ± SD or SEM of independent experiments as indicated in figure legends. Significant differences were identified using one-way or two-way ANOVA and appropriate post-hoc comparison tests, as indicated in figure legends. Differences are indicated by ∗/† = *p* < 0.05, ∗∗/†† = *p* < 0.01, ∗∗∗ = *p* < 0.0 1, ∗∗∗∗ = *p* < 0.0001.

## References

[1] Gentek R., Ghigo C., Hoeffel G., Bulle M.J., Msallam R., Gautier G., Launay P., Chen J., Ginhoux F., Bajénoff M. (2018). Hemogenic Endothelial Fate Mapping Reveals Dual Developmental Origin of Mast Cells. Immunity.

[2] Wang X., Yang L., Wang Y.C., Xu Z.R., Feng Y., Zhang J., Wang Y., Xu C.R. (2020). Comparative analysis of cell lineage differentiation during hepatogenesis in humans and mice at the single-cell transcriptome level. Cell Res..

[3] West P.W., Bahri R., Garcia-Rodriguez K.M., Sweetland G., Wileman G., Shah R., Montero A., Rapley L., Bulfone-Paus S. (2021). Interleukin-33 Amplifies Human Mast Cell Activities Induced by Complement Anaphylatoxins. Front. Immunol..

[4] McNeil B.D., Pundir P., Meeker S., Han L., Undem B.J., Kulka M., Dong X. (2015). Identification of a mast-cell-specific receptor crucial for pseudo-allergic drug reactions. Nature.

[5] Tatemoto K., Nozaki Y., Tsuda R., Konno S., Tomura K., Furuno M., Ogasawara H., Edamura K., Takagi H., Iwamura H. (2006). Immunoglobulin E-independent activation of mast cell is mediated by Mrg receptors. Biochem. Biophys. Res. Commun..

[6] Gour N., Dong X. (2024). The MRGPR family of receptors in immunity. Immunity.

[7] Varricchi G., Pecoraro A., Loffredo S., Poto R., Rivellese F., Genovese A., Marone G., Spadaro G. (2019). Heterogeneity of human mast cells with respect to MRGPRX2 receptor expression and function. Front. Cell. Neurosci..

[8] Li Z., Liu S., Xu J., Zhang X., Han D., Liu J., Xia M., Yi L., Shen Q., Xu S. (2018). Adult Connective Tissue-Resident Mast Cells Originate from Late Erythro-Myeloid Progenitors. Immunity.

[9] Subramanian H., Gupta K., Guo Q., Price R., Ali H. (2011). Mas-related gene X2 (MrgX2) is a novel G protein-coupled receptor for the antimicrobial peptide LL-37 in human mast cells: resistance to receptor phosphorylation, desensitization, and internalization. J. Biol. Chem..

[10] Subramanian H., Gupta K., Lee D., Bayir A.K., Ahn H., Ali H. (2013). β-Defensins activate human mast cells via Mas-related gene X2. J. Immunol..

[11] Al Hamwi G., Riedel Y.K., Clemens S., Namasivayam V., Thimm D., Müller C.E. (2022). MAS-related G protein-coupled receptors X (MRGPRX): Orphan GPCRs with potential as targets for future drugs. Pharmacol. Ther..

[12] Babina M., Wang Z., Roy S., Guhl S., Franke K., Artuc M., Ali H., Zuberbier T. (2021). MRGPRX2 Is the Codeine Receptor of Human Skin Mast Cells: Desensitization through β-Arrestin and Lack of Correlation with the FcεRI Pathway. J. Invest. Dermatol..

[13] Subramanian H., Gupta K., Ali H. (2016). Roles of Mas-related G protein-coupled receptor X2 on mast cell-mediated host defense, pseudoallergic drug reactions, and chronic inflammatory diseases. J. Allergy Clin. Immunol..

[14] Hägermark O., Hökfelt T., Pernow B. (1978). Flare and Itch Induced by Substance P in Human Skin. J. Invest. Dermatol..

[15] Green D.P., Limjunyawong N., Gour N., Pundir P., Dong X. (2019). A Mast-Cell-Specific Receptor Mediates Neurogenic Inflammation and Pain. Neuron.

[16] Meixiong J., Anderson M., Limjunyawong N., Sabbagh M.F., Hu E., Mack M.R., Oetjen L.K., Wang F., Kim B.S., Dong X. (2019). Activation of Mast-Cell-Expressed Mas-Related G-Protein-Coupled Receptors Drives Non-histaminergic Itch. Immunity.

[17] Nattkemper L.A., Tey H.L., Valdes-Rodriguez R., Lee H., Mollanazar N.K., Albornoz C., Sanders K.M., Yosipovitch G. (2018). The Genetics of Chronic Itch: Gene Expression in the Skin of Patients with Atopic Dermatitis and Psoriasis with Severe Itch. J. Invest. Dermatol..

[18] Manorak W., Idahosa C., Gupta K., Roy S., Panettieri R., Ali H. (2018). Upregulation of Mas-related G Protein coupled receptor X2 in asthmatic lung mast cells and its activation by the novel neuropeptide hemokinin-1. Respir. Res..

[19] Navinés-Ferrer A., Serrano-Candelas E., Lafuente A., Muñoz-Cano R., Martín M., Gastaminza G. (2018). MRGPRX2-mediated mast cell response to drugs used in perioperative procedures and anaesthesia. Sci. Rep..

[20] Weitzmann A., Naumann R., Dudeck A., Zerjatke T., Gerbaulet A., Roers A. (2020). Mast Cells Occupy Stable Clonal Territories in Adult Steady-State Skin. J. Invest. Dermatol..

[21] Roy S., Chompunud Na Ayudhya C., Thapaliya M., Deepak V., Ali H. (2021). Multifaceted MRGPRX2: New insight into the role of mast cells in health and disease. J. Allergy Clin. Immunol..

[22] West P.W., Tontini C., Atmoko H., Kiss O., Garner T., Bahri R., Warren R.B., Griffiths C.E.M., Stevens A., Bulfone-Paus S. (2023). Human Mast Cells Upregulate Cathepsin B, a Novel Marker of Itch in Psoriasis. Cells.

[23] Chéret J., Piccini I., Gherardini J., Ponce L., Bertolini M., Paus R. (2022). Sensory Reinnervation of Human Skin by Human Neural Stem Cell‒Derived Peripheral Neurons Ex Vivo. J. Invest. Dermatol..

[24] Fujisawa D., Kashiwakura J.-I., Kita H., Kikukawa Y., Fujitani Y., Sasaki-Sakamoto T., Kuroda K., Nunomura S., Hayama K., Terui T. (2014). Expression of Mas-related gene X2 on mast cells is upregulated in the skin of patients with severe chronic urticaria. J. Allergy Clin. Immunol..

[25] Babina M., Wang Z., Artuc M., Guhl S., Zuberbier T. (2018). MRGPRX2 is negatively targeted by SCF and IL-4 to diminish pseudo-allergic stimulation of skin mast cells in culture. Exp. Dermatol..

[26] Nieber K., Baumgarten C., Rathsack R., Furkert J., Laake E., Müller S., Kunkel G. (1993). Effect of azelastine on substance P content in bronchoalveolar and nasal lavage fluids of patients with allergic asthma. Clin. Exp. Allergy.

[27] Tomaki M., Ichinose M., Miura M., Hirayama Y., Yamauchi H., Nakajima N., Shirato K. (1995). Elevated Substance P Content in Induced Sputum from Patients with Asthma and Patients with Chronic Bronchitis. Am. J. Respir. Crit. Care Med..

[28] Plum T., Wang X., Rettel M., Krijgsveld J., Feyerabend T.B., Rodewald H.R. (2020). Human Mast Cell Proteome Reveals Unique Lineage, Putative Functions, and Structural Basis for Cell Ablation. Immunity.

[29] Gong Y., Johnsson A.K., Säfholm J., Al-Ameri M., Sachs E., Vali K., Nilsson G., Rönnberg E. (2024). An optimized method for IgE-mediated degranulation of human lung mast cells. Front. Immunol..

[30] Joos G., Pauwels R., Van Der Straeten M. (1987). Effect of inhaled substance P and neurokinin A on the airways of normal and asthmatic subjects. Thorax.

[31] Alkanfari I., Gupta K., Jahan T., Ali H. (2018). Naturally Occurring Missense MRGPRX2 Variants Display Loss of Function Phenotype for Mast Cell Degranulation in Response to Substance P, Hemokinin-1, Human β-Defensin-3, and Icatibant. J. Immunol..

[32] Gaudenzio N., Sibilano R., Marichal T., Starkl P., Reber L.L., Cenac N., McNeil B.D., Dong X., Hernandez J.D., Sagi-Eisenberg R. (2016). Different activation signals induce distinct mast cell degranulation strategies. J. Clin. Invest..

[33] Al Hamwi G., Namasivayam V., Büschbell B., Gedschold R., Golz S., Müller C.E. (2024). Proinflammatory chemokine CXCL14 activates MAS-related G protein-coupled receptor MRGPRX2 and its putative mouse ortholog MRGPRB2. Commun. Biol..

[34] Azimi E., Reddy V.B., Pereira P.J.S., Talbot S., Woolf C.J., Lerner E.A. (2017). Substance P activates Mas-related G protein–coupled receptors to induce itch. J. Allergy Clin. Immunol..

[35] Amatya B., Nordlind K., Wahlgren C.F. (2010). Responses to intradermal injections of substance P in psoriasis patients with pruritus. Skin Pharmacol. Physiol..

[36] Metz M., Krull C., Hawro T., Saluja R., Groffik A., Stanger C., Staubach P., Maurer M. (2014). Substance P is upregulated in the serum of patients with chronic spontaneous urticaria. J. Invest. Dermatol..

[37] West P.W., Bulfone-Paus S. (2022). Mast cell tissue heterogeneity and specificity of immune cell recruitment. Front. Immunol..

[38] Collington S.J., Williams T.J., Weller C.L. (2011). Mechanisms underlying the localisation of mast cells in tissues. Trends Immunol..

[39] Halova I., Draberova L., Draber P. (2012). Mast cell chemotaxis - chemoattractants and signaling pathways. Front. Immunol..

[40] Weller C.L., Collington S.J., Brown J.K., Miller H.R.P., Al-Kashi A., Clark P., Jose P.J., Hartnell A., Williams T.J. (2005). Leukotriene B4, an activation product of mast cells, is a chemoattractant for their progenitors. J. Exp. Med..

[41] Kuehn H.S., Jung M.Y., Beaven M.A., Metcalfe D.D., Gilfillan A.M. (2011). Distinct PGE2-responder and non-responder phenotypes in human mast cell populations: “all or nothing” enhancement of antigen-dependent mediator release. Immunol. Lett..

[42] Weller C.L., Collington S.J., Hartnell A., Conroy D.M., Kaise T., Barker J.E., Wilson M.S., Taylor G.W., Jose P.J., Williams T.J. (2007). Chemotactic action of prostaglandin E2 on mouse mast cells acting via the PGE2 receptor 3. Proc. Natl. Acad. Sci. USA.

[43] Jung I.D., Lee H.S., Lee H.Y., Choi O.H. (2009). FcεRI-mediated mast cell migration: Signaling pathways and dependence on cytosolic free Ca^2+^ concentration. Cell. Signal..

[44] Guo Y., Ollé L., Proaño-Pérez E., Aparicio C., Guerrero M., Muñoz-Cano R., Martín M. (2023). MRGPRX2 signaling involves the Lysyl-tRNA synthetase and MITF pathway. Front. Immunol..

[45] Okabe T., Hide M., Koro O., Nimi N., Yamamoto S. (2001). The release of leukotriene B4 from human skin in response to substance P: evidence for the functional heterogeneity of human skin mast cells among individuals. Clin. Exp. Immunol..

[46] Okabe T., Hide M., Hiragun T., Morita E., Koro O., Yamamoto S. (2006). Bone marrow derived mast cell acquire responsiveness to substance P with Ca(2+) signals and release of leukotriene B(4) via mitogen-activated protein kinase. J. Neuroimmunol..

[47] Asahara M., Ito N., Hoshino Y., Sasaki T., Yokomizo T., Nakamura M., Shimizu T., Yamada Y. (2022). Role of leukotriene B4 (LTB4)-LTB4 receptor 1 signaling in post-incisional nociceptive sensitization and local inflammation in mice. PLoS One.

[48] Martin H.A., Basbaum A.I., Goetzl E.J., Levine J.D. (1988). Leukotriene B4 decreases the mechanical and thermal thresholds of C-fiber nociceptors in the hairy skin of the rat. J. Neurophysiol..

[49] Zinn S., Sisignano M., Kern K., Pierre S., Tunaru S., Jordan H., Suo J., Treutlein E.M., Angioni C., Ferreiros N. (2017). The leukotriene B4 receptors BLT1 and BLT2 form an antagonistic sensitizing system in peripheral sensory neurons. J. Biol. Chem..

[50] Chéret J., Ponce L., Le Gall-Ianotto C., Bertolini M., Paus R. (2021). Re-innervation of human skin by rat dorsal root ganglia permits to study interactions between sensory nerve fibres and native human dermal mast cells ex vivo. Exp. Dermatol..

[51] Andoh T., Katsube N., Maruyama M., Kuraishi Y. (2001). Involvement of leukotriene b4 in substance p-induced itch- associated response in mice. J. Invest. Dermatol..

[52] Bahri R., Custovic A., Korosec P., Tsoumani M., Barron M., Wu J., Sayers R., Weimann A., Ruiz-Garcia M., Patel N. (2018). Mast cell activation test in the diagnosis of allergic disease and anaphylaxis. J. Allergy Clin. Immunol..

[53] Groot Kormelink T., Arkesteijn G.J.A., van de Lest C.H.A., Geerts W.J.C., Goerdayal S.S., Altelaar M.A.F., Redegeld F.A., Nolte-’t Hoen E.N.M., Wauben M.H.M. (2016). Mast Cell Degranulation Is Accompanied by the Release of a Selective Subset of Extracellular Vesicles That Contain Mast Cell–Specific Proteases. J. Immunol..

[54] Grützkau A., Smorodchenko A., Lippert U., Kirchhof L., Artuc M., Henz B.M. (2004). LAMP-1 and LAMP-2, but not LAMP-3, are reliable markers for activation-induced secretion of human mast cells. Cytometry A..

[55] Lauria G., Hsieh S.T., Johansson O., Kennedy W.R., Leger J.M., Mellgren S.I., Nolano M., Merkies I.S.J., Polydefkis M., Smith A.G. (2010). European Federation of Neurological Societies/Peripheral Nerve Society Guideline on the use of skin biopsy in the diagnosis of small fiber neuropathy. Report of a joint task force of the European Federation of Neurological Societies and the Peripheral Nerve Society. Eur. J. Neurol..

