# Supplementary material for: The MRGPRX2-substance P pathway regulates mast cell migration
Source: iScience. 2024 Sep 16;27(10):110984. doi: 10.1016/j.isci.2024.110984 (PMC11492034; doi:10.1016/j.isci.2024.110984)
Supplement: Document S1. Figures S1–S4 and Table S1 [file mmc1.pdf]

iScience, Volume *27*

Supplemental information

The MRGPRX2-substance P pathway regulates mast cell migration

Peter W. West, Jérémy Chéret, Rajia Bahri, Orsolya Kiss, Zining Wu, Colin H. Macphee, and Silvia Bulfone-Paus

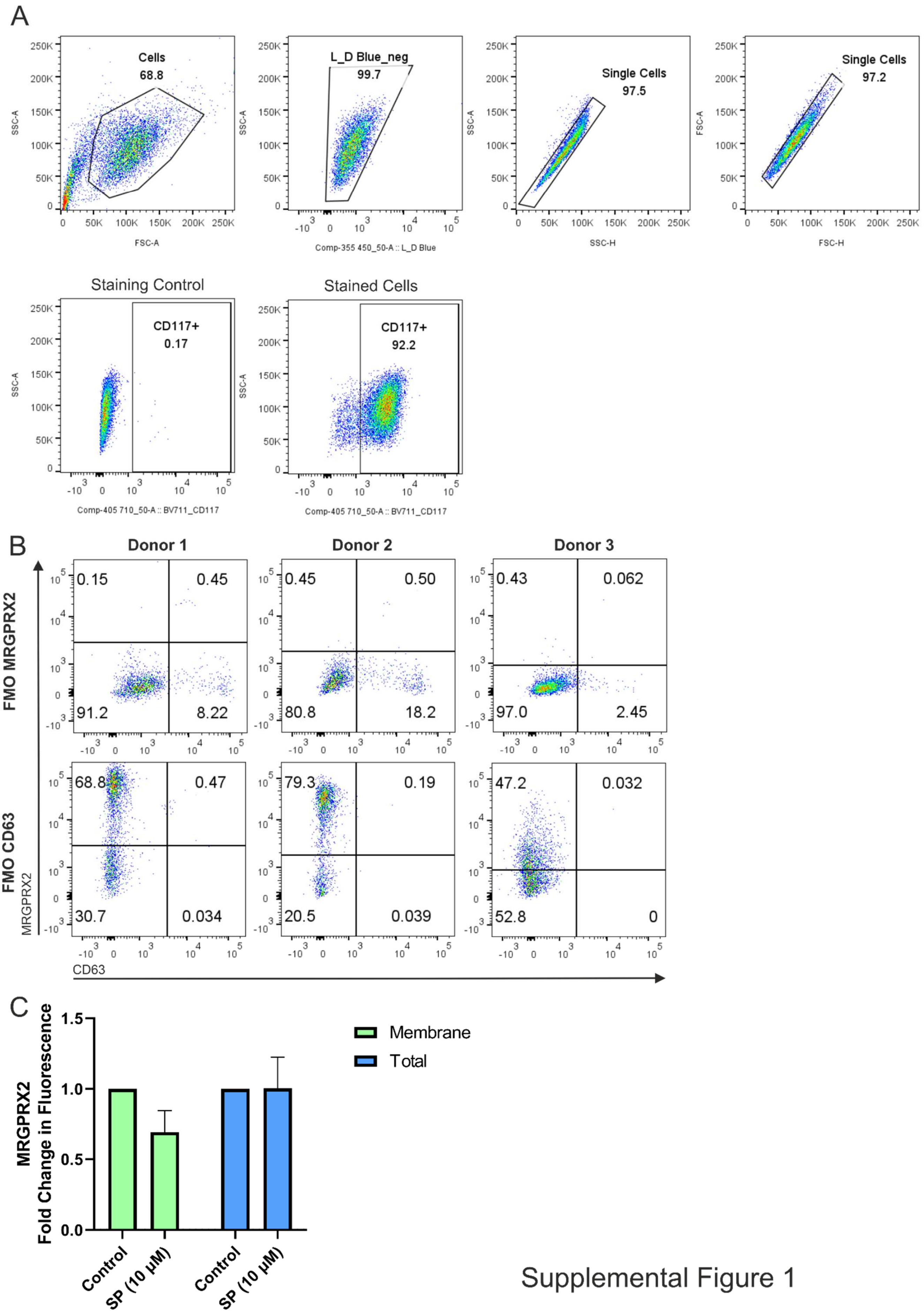

Supplemental Figure 1

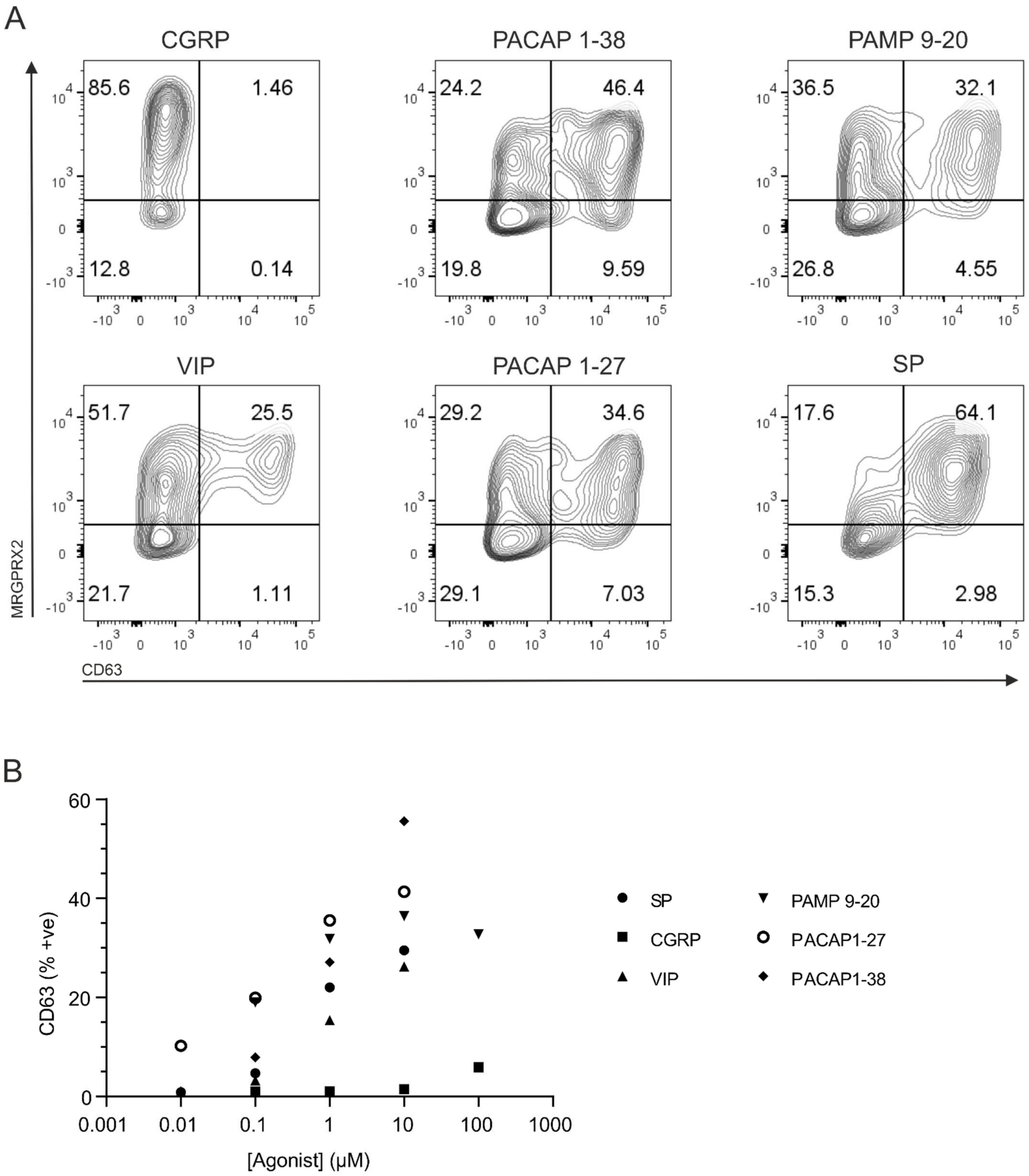

Supplemental Figure 2

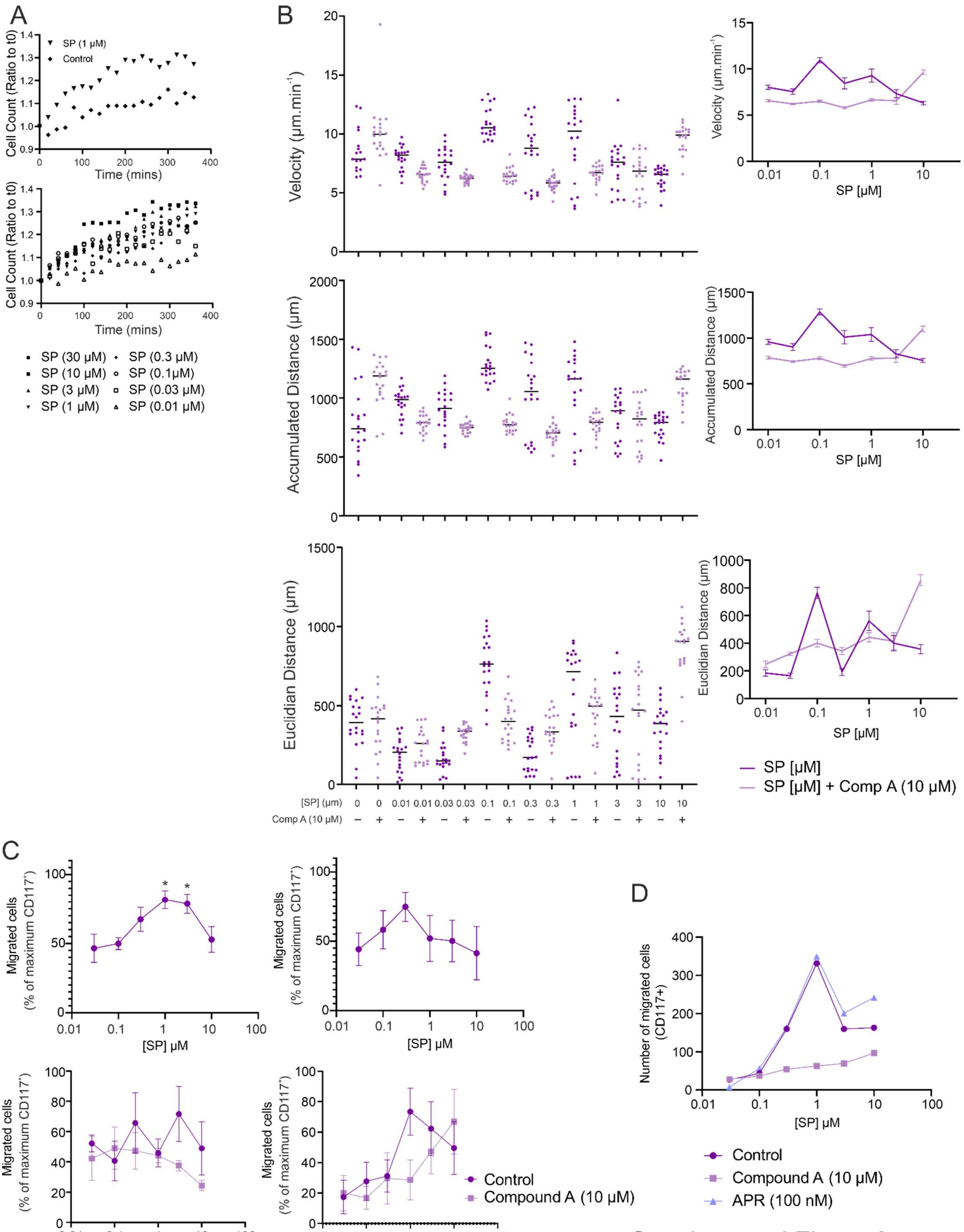

Supplemental Figure 3

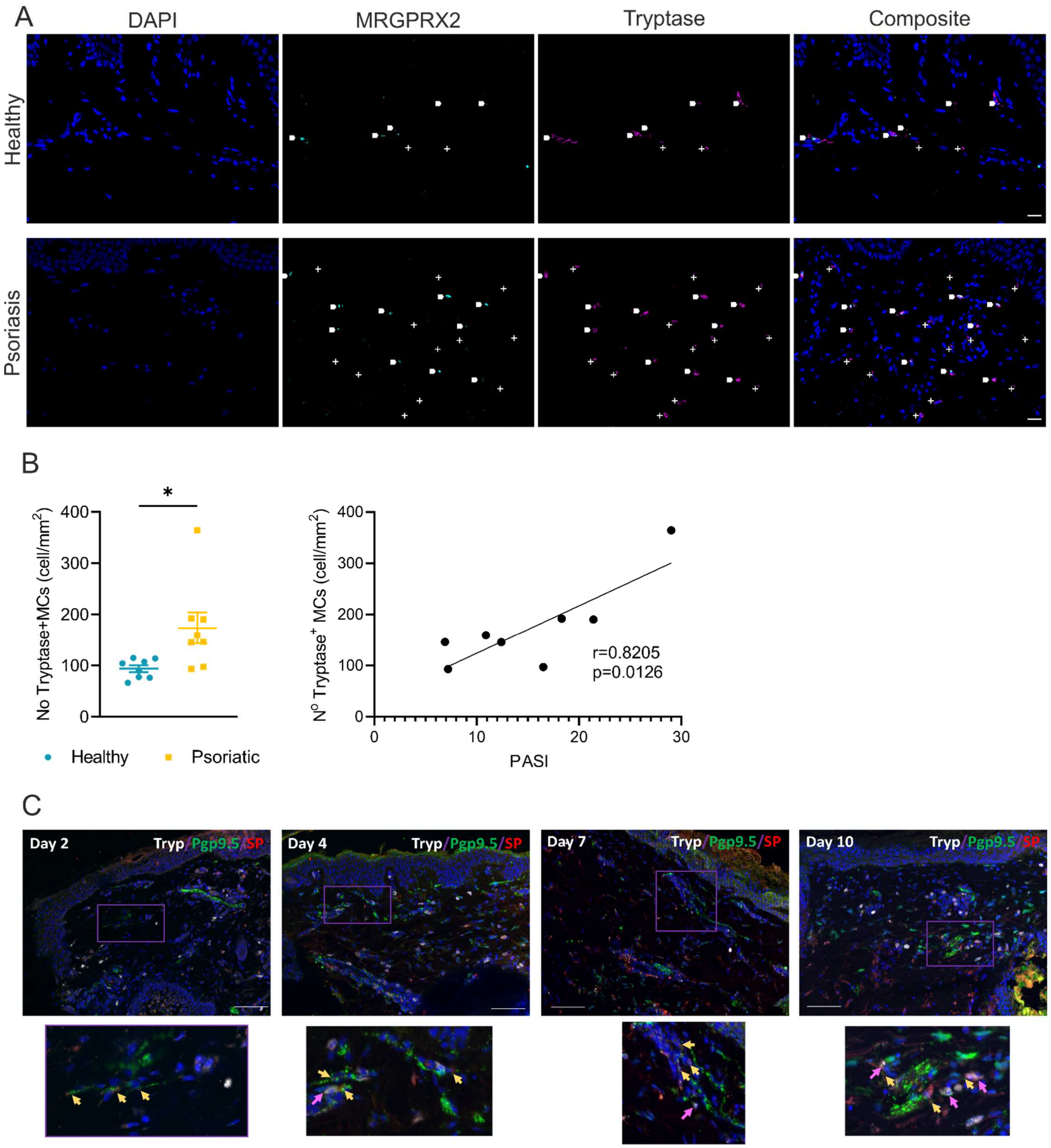

Supplemental Figure 4

Supplemental Figure Legends

Supplemental Figure 1. Example gating strategy for mast cells. Related to Figure 1

(A) Gating as shown for live cell population based on forward/side scatter, exclusion of dead cells by live/dead blue stain, single cells based on both forward and side scatter height and area (SSC/FSC-A/H). Each marker was gated based on Fluorescence Minus One (FMO) controls with example for CD117. (B) three FMO controls for MRGPRX2 and CD63 shown. (C) Fold change in membrane alone or total (membrane + intracellular) MRGPRX2 specific fluorescence with or without stimulation with SP (10 µM) for 3 minutes. Data are mean ± S.E.M. of n=3 donors.

Supplemental Figure 2. Putative MRGPRX2 ligand activation of MRGPRX2 positive cells. Related to Figure 2

(A) Gating showing degranulation, as measured by CD63 externalisation (x-axis), vs MRGPRX2 expression (y-axis) after 15 mins of treatment with labelled agonists at 1µM (PACAP1-38, PACAP1-27, PAMP9-20, VIP) or 10 µM (CGRP, SP) concentration. Percentage of cells in each quadrant is shown.

(B) Degranulation of mast cells in response to agonists (0.01-100 µM) after 15 minutes of application, as shown. Data are n=1 of a mix of 2 donors.

Supplemental Figure 3. Mast Cells migrate towards SP in an MRGPRX2 and concentration dependent manner. Related to Figure 3

(A) Mast cell migration towards pores in the upper chamber of an IncuCyte™ clearview cell migration plate. Increase in cells counted moving towards central pores of the plate over 6 hour recording (360 mins). Comparison between 1 µM SP and control (upper panel) and for each SP concentration (lower panel). Data are n=1.

(B) Cell migration recorded in a flat bottom 96 well plate where SP was added to the side of the well. Individual cell tracking carried out using the ibidi chemotaxis and migration tool based on 6 hour recording. For each track the velocity (upper panels) accumulated (total) distance, and Euclidian distance (straight distance from start to finish point) was measured. Mean data are presented in right hand panels. Data are for n=19-20 cell tracks per condition.

(C) Chemotaxis of immature (left) and mature (right) mast cells in response to SP (upper panels). Data are mean ± SEM of n=9 (immature) and n=7 (mature). Inhibition of chemotaxis in response to PAMP 9-20 (lower panels) by compound A (10 µM). Data are mean ± SEM of n=4 separate donors. * = $p<0.05$ (Friedman test).

(D) Inhibition of SP induced chemotaxis of immature mast cells in response to 0.03-10 SP in the presence of vehicle control, 10 µM Compound A, or 100 nM Aprepitant. Date are n=1.

Supplemental Figure 4. Images of mast cells in skin sections and further analysis. Related to Figure 4

(A) Representative images of mast cells (MCs) in skin sections showing single channel and composite images.

(B) Density of tryptase+ MCs in healthy and psoriasis skin sections and correlation between tryptase+ MC density and PASI. Data are mean ± SEM of n=6 donors (unpaired t-test). Correlation was determined by Pearson's statistic.

(C) Representative images of human skin biopsies after 2, 4, 7 & 10 days of co-culture with sensory neurons and stained with anti-sera to PGP9.5, tryptase and substance P and counterstained with DAPI. Scale bar = 100 µm. Yellow arrows: PGP9.5+SP+ nerve fibres; Pink arrows: Mast cells in close contact with PGP9.5+SP+ nerve fibre.

Supplemental Table 1. Subject demographics used for MRGPRX2 immunohistochemistry.

	**Psoriasis**	**Healthy**	**p value**
**N**	6	6	
**Age (mean ± SD)**	51 ± 22	57 ± 22	0.6767
**Male:Female**	2:4	3:3	>0.9999
